# The clinical features and impact of SARS-CoV-2/COVID-19 infection in children with Cystic Fibrosis (CF): A Qatari experience

**DOI:** 10.5339/qmj.2023.19

**Published:** 2023-12-12

**Authors:** Sara G Hamad, Hiba Kammouh, Mohammed Alamri, Khalid Zahraldin

**Affiliations:** ^1^Section of Pediatric Pulmonology, Department of Pediatrics, Hamad Medical Corporation, Doha, Qatar Email: shamad3@hamad.qa ORCID iD: 0000-0002-8292-2011; ^2^Section of Pediatric Emergency, Department of Pediatrics, Hamad Medical Corporation, Doha, Qatar; ^3^Weill Cornell Medicine, Doha, Qatar Email: shamad3@hamad.qa ORCID iD: 0000-0002-8292-2011

**Keywords:** CF: Cystic Fibrosis, COVID-19: Coronavirus disease 2019, SARS-CoV-2: severe acute respiratory syndrome coronavirus 2

## Abstract

Background: SARS-CoV-2 in children with cystic fibrosis (CF) has been reported to cause mild illness without pre-existing severe lung disease. This review described the clinical presentation and course of COVID-19 infection in children with CF in Qatar.

Methods: The pediatric CF registry of 51 patients in Qatar was reviewed for COVID-19 cases from February 2020 to February 2022. Demographics, vaccination status, symptoms, and course were reviewed. Data were expressed as median, range, frequencies, and percentages.

Results: The study included eight patients with CF below 18 years of age infected with COVID-19. The incidence of COVID-19 in children with CF was 15.7%. The median age was 11 (2-18) years. Half of the cohort were males. Seven patients were pancreatic sufficient (I1234V mutation), and one was pancreatic insufficient (3129del4 mutation). The median baseline FEV1 was 91 (78-107%) predicted. None had received CFTR modulators or undergone a lung transplant. Three patients were vaccinated before their infections. Two of them were asymptomatic. Six patients (75%) had a cough and flu-like symptoms. Three patients had a fever. Two patients were hospitalized due to pulmonary exacerbation; both had mild CF-lung disease. None required respiratory support.

Conclusion: We report a favorable outcome of COVID-19 infection in children with CF, similar to published international studies. Our findings are attributable to the community-dominant milder CFTR mutation, precautionary measures, and causative COVID-19 strain. More longitudinal data are needed to study these factors as potential protective mechanisms.

## Introduction

Cystic fibrosis (CF) is an inherited autosomal recessive disease caused by mutations in the cystic fibrosis transmembrane conductance regulator (CFTR) protein.^1^ Over two thousand mutations have been identified, with F508del being the most common mutation.^1^ These mutations lead to multi-systematic disease involving pulmonary, gastrointestinal, reproductive, and endocrine systems.^2^

The prevalence of CF in the Middle East is estimated to be 1 in 30,000–50,000.^3,4^ In the state of Qatar, the CF population is peculiar for the dominance of CFTR I1234V mutation, especially among Bedouin tribes^5^ compared to the Arab region and worldwide. Nevertheless, other rare mutations were described in a few reports among the non-Qatari population residing in Qatar.^6–9^

The presence of homozygous CFTR I1234V mutation was first described by Abdul Wahab et al. in patients with variable disease manifestations. Those patients were from 17 families from the same Bedouin tribe, with a high consanguinity rate of 96.6%.^10^

The reported Qatari pediatric population had mild to moderate respiratory symptoms and was characterized by pancreatic sufficiency. In comparison, children with CF of non-Arabic Asian descent had more severe respiratory symptoms with an earlier occurrence of Pseudomonas aeruginosa colonization and pancreatic insufficiency.^11^

Respiratory viruses cause pulmonary exacerbations and significant morbidity in patients with CF^1,12,13^ due to impaired innate immune response of CF airway epithelia to respiratory viruses.^14–16^ This predisposition was particularly evident during the H1N1 pandemic in 2019, which resulted in significant morbidity and mortality among CF patients.^17,18^

The pandemic of severe acute respiratory syndrome coronavirus 2 (SARS-CoV-2) has caused a significant global health impact.^19,20^ Compared with COVID-19 infection in adults, COVID-19 infection in the pediatric population is usually asymptomatic or causes mild symptoms.^21,22^ Also, certain risk factors for severe disease were identified in adults affected by COVID-19 infection.^23^ Few studies and case reports have reported an unexpectedly favorable clinical outcome of COVID-19 in CF patients.^24–26^ Altered intracellular processes were displayed by CF airway epithelial cells during host defense against SARS-CoV-2 and viral replication. These molecular processes are hypothesized as protective factors of CF patients against COVID-19.^27^

The clinical course and outcome of COVID-19 infection among children with CF in our community must be better characterized and have yet to be previously reported. This signifies a critical knowledge gap and an essential step in preventing and managing these children during the COVID-19 pandemic. In this descriptive study, we describe the incidence, clinical features, and outcomes of COVID-19 infection in children with CF in Qatar to provide precise information about the clinical course and identify any potential risk factors for severe respiratory complications of COVID-19 disease among these children.

## Methods

We retrospectively reviewed medical electronic charts at Hamad Medical Corporation in Qatar. Our Pediatric CF registry, which contains 51 patients, was reviewed for positive COVID-19 cases from February 2020 to the end of February 2022. All children (below 18 years) with a confirmed diagnosis of CF with evidence of serologically confirmed COVID-19 infection were included in the study. The confirmation of COVID-19 infection was established by Polymerase chain reaction (PCR) or Rapid antigen (Ag) testing of nasal swabs. Patients with cystic fibrosis above 18 years of age and those without serologically confirmed COVID-19 infection were excluded.

Demographics, presenting symptoms, vaccination status, and clinical course data were collected. Quantitative data were presented as median and range, while qualitative data were demonstrated as frequencies and percentages (%). No missing data was identified during the data collection. The Institutional Review Board of the Medical Research Center at Hamad Medical Corporation (MRC-01-22-604) approved the study on the 17^th^ of October, 2022.

## Results

Eight patients with CF < 18 years of age were infected with serologically confirmed COVID-19. The diagnosis was made by obtaining nasopharyngeal swabs for either polymerase chain reaction (PCR) or rapid antigen testing (Ag). The incidence of COVID-19 among the CF pediatric population was 15 per 100 persons from 2020 to 2022. The median age at infection was 11 (2-18) years. Half of the patients were males. The median body mass index Z-score was 0.08 (-1.95 – 1.64).

Seven patients have been pancreatic sufficient (CFTR I1234V mutation), and one is pancreatic insufficient (3129del4 mutation). Seven patients had baseline pulmonary function testing. The median baseline FEV1 was 91 (78-107%) predicted.

Before the COVID-19 infection, six patients had been colonized with Methicillin-sensitive staphylococcus aureus (MSSA), which was isolated from their sputum culture. One of them had concurrent pseudomonas aeruginosa (PsA) colonization, and another patient had a concurrent non-tuberculosis mycobacterium (NTM) isolation. The remaining two patients had Methicillin-resistant staphylococcus aureus (MRSA).

Six patients in our cohort had baseline chest computed tomography (CT) before COVID-19 infection. Three of them had central cylindrical bronchiectasis. Two had central cystic bronchiectasis. One patient had mildly dilated bronchi with no significant bronchiectasis. All patients were maintained on nebulized sodium chloride at 7%, except for one patient who has been on nebulized sodium chloride at 3%. Two patients were receiving dornase alpha treatment before COVID-19 acquisition. None of the patients had been receiving Azithromycin as an anti-inflammatory therapy before or during the COVID-19 infection. None of the patients had received CFTR modulators or underwent lung transplants. None had CF-related diabetes (CFRD). Before their infections, three patients were vaccinated with mRNA COVID-19 vaccines (Pfizer-BioNTech®). The remaining patients ’characteristics are summarized in Table 1.

Most infections (6 out of 8 patients) were encountered during the COVID-19 wave (Omicron variant) 2022. Two of the patients were asymptomatic. In contrast, six patients (75%) had cough and flu-like symptoms. Three patients had a fever. None reported dyspnea, hemoptysis, myalgia, or gastrointestinal symptoms.

Two patients were hospitalized due to pulmonary exacerbation, and both had mild CF-lung disease with pancreatic sufficiency, including the only patient with PsA. They were aged 2 and 11 years. Both patients presented with symptoms of fever, cough, and upper respiratory tract infection. Both patients had concurrent infection with group-A streptococcus in one patient and parainfluenza virus in addition to chlamydia pneumonia in the other. None required respiratory support or intensive care admission. The median duration of the hospitalization was seven days (3-10) days. All patients, including those hospitalized, completed a quarantine period of 10-14 days at home or a Quarantine Facility according to Qatar-CDC recommendations during the concurrent COVID-19 wave. Three patients required oral antibiotics during their illness. None of the patients received anti-viral therapy or corticosteroids. Follow-up spirometry post-COVID-19 infection was not feasible and was not performed due to COVID-19 restrictions.

Post-COVID-19 infection, four patients had MRSA isolated from their sputum culture. One patient had acquired new PsA isolation. In addition, two patients had NTM isolated, and one received the full NTM treatment protocol, which consisted of oral rifampin, azithromycin, ethambutol, clofazimine, and nebulized amikacin for one year. It is worth mentioning that in the patients who had PsA and NTM before the COVID-19 infection, their sputum culture did not grow these organisms on their routine follow-up cultures.

Only two patients had follow-up CT chest. Both chest CTs showed worsening bronchiectasis changes. However, one of them had a concurrent NTM infection. All the patients, including the two hospitalized patients, fully recovered with no residual symptoms. Table 2 delineates the summary of individual patient’s presentation and outcome.

## Discussions

This work represents the first study to describe the incidence of COVID-19 infection in our pediatric CF population. It also represents the only study that describes the clinical course of children with CF with CFTR I1234V mutation.

In Qatar, various precautionary measures were implemented in the early stages of the COVID-19 pandemic^28^ to limit the spread of COVID-19 infection. These measures addressed the general population and focused on high-risk patients, including cystic fibrosis.^29^ The incidence of COVID-19 among our CF pediatric population was 15 per 100 persons from 2020 to 2022. However, no data about the incidence among the general pediatric population in Qatar has been published.

Moreover, there was no reported data in the Arab Gulf region that reflects the pediatric CF population. However, Alyazidi et al.^30^ reported COVID-19 infection in three children with CF who required hospitalization during the first year of the COVID-19 wave in three main pediatric hospitals in Oman. Only one patient with moderate-severe CF-related lung disease required respiratory support with non-invasive ventilation.

The CF Registry Global Harmonization Group published an initial report of COVID-19 cases and described a spectrum of clinical severity, with most adult cases being mild or moderate.^27^ However, the updated report recorded seven deaths.^31^

Our results were comparable to the largest pediatric international results, which reported the favorable clinical course and outcomes of SARS-CoV-2 infection in 105 children with CF who do not have pre-existing severe lung disease.^32^ In this study, only 24 children required hospitalization. Those patients had lower lung function and reduced body mass index Z-scores. Only one child died due to non-COVID-19 complications.

The published data suggested that CF, unexpectedly, may represent an advantage in COVID-19 infection.^33^ These suggestions were based on various studies on molecular basis, cytokines, and CF medications. The innate immune response of airway epithelia to respiratory viruses was impaired in patients with CF.^14–16^ Nevertheless, CF airway epithelial cells were hypothesized to display different intracellular host defenses and viral replication.^27^

Elevated interleukin-6 (IL-6) levels were linked with severe COVID-19 disease course and mortality.^34,35^ Nevertheless, the airway epithelia in CF patients had reduced IL-6 levels suggesting a protective effect against severe SARS-CoV-2 infection.^34^ Marcinkiewicz et al. reported high interleukin-8 (IL-8) levels and extremely low IL-6 and interleukin-10 (IL-10) levels in the sputum samples of 39 patients with advanced CF and chronic Pseudomonas aeruginosa infection. Whereas serological IL-6 levels were not affected.^34^

Angiotensin-converting enzyme 2 (ACE2) was found to be the host cell binding- and entry-receptor of SARS-CoV-2.^36^ The SARS-CoV-2 infection causes ACE2 transcriptional downregulation, leading to increased synthesis of angiotensin II,^37^ which potentially leads to further lung injury.^38^

In CF, the effect of ACE biallelic polymorphism was studied in 180 patients with CF. The study reported that CF patients with biallelic deletion of ACE were associated with earlier clinical symptoms and a higher risk of lung deterioration than patients with ACE biallelic or monoallelic insertion polymorphisms.^39^ The effect of ACE polymorphisms and ACE2 downregulation exhibited in patients with CF may be attributed to the variability of the COVID-19 course in those patients.^40^

The presence of thick secretions and existing microbiota in the airways of patients with CF may also possess protective effects against viral infection.^41^ In addition, the elevated autophagy induction in patients with CF may also enhance the anti-viral response as an additional protective mechanism.^27,42^

Other suggested protective effects against COVID-19 were CF medications.^33^ The effect of inhaled dornase alfa, commonly used to manage secretions in CF patients, was studied. The proposed protective mechanism for COVID-19 infection is based on the clearance of neutrophil extracellular traps.^43^ In our study, only two patients were on inhaled dornase alfa and had mild symptoms.

The effect of Azithromycin has also been described in a few studies where it has been either administered as a treatment for COVID-19 or has been given as maintenance therapy for patients with CF.^32,41,44,45^ The proposed mechanism was immune modulation and weak anti-viral effects.^46,47^

Azithromycin has shown immunomodulatory effects on innate and adaptive immunity in cystic fibrosis and other inflammatory respiratory diseases, as well as its well-established antibacterial effects. However, azithromycin had shown no clinical immunomodulatory effects in the context of COVID-19 infection.^48^

Even though the COVID-19 outcome is favorable in most patients with CF, independent risk factors for the severe outcome of COVID-19 infection in CF patients were identified. Those included lung transplantation, CFRD, and moderate to severe lung disease.^49^ Another study reported additional risk factors such as the previous need for oxygen therapy, severe lung function impairment, and CF with pancreatic insufficiency.^50^ Fortunately, those risk factors were absent in the reported cases in our study, except for one patient who had pancreatic insufficiency but had mild disease.

It is worth noting that respiratory symptoms remain the most common presentation in our study. Children who were vaccinated had minimal symptoms or not at all. Both hospitalized patients had a concurrent infection which may have partially contributed to their illness.

The isolation of MRSA and PsA, post-COVID-19 infection in five patients, might be attributed to average disease progress or lack of early detection and treatment usually done as part of routine follow-up clinic visits interrupted during the COVID-19 pandemic.

We acknowledge the limitations of our study. The study is retrospective and has a small sample size. It includes patients based on self-/parent-reporting or random screening after contact exposure. Inevitably, asymptomatic cases were not registered, which may have underestimated the incidence. Three of our patients were diagnosed using rapid Ag testing and not confirmed by PCR.

The missing objective follow-up data, such as spirometry post-COVID-19 infection, was expected due to the implementation of telemedicine amid the COVID-19 pandemic. Nonetheless, our study shed light on the incidence and clinical course of COVID-19 in children with CF with CFTR I1234V mutation that is distinctive to our region. This would help clinicians provide accurate advice for preventing and managing these children with CF in our community. In addition, it would help clinicians to recognize potential or modifiable risk factors for severe disease.

## Conclusions

We report a favorable outcome of COVID-19 infection in children with CF, comparable to published international studies. Our findings may be attributed to the community-dominant milder CFTR mutation, precautionary measures; awareness and vaccination campaigns; abundance of resources, and causative COVID-19 strain. However, vaccinations and preventive measures remain essential in preventing COVID-19 in children with CF. Further longitudinal data are needed to assess the long-term effects of COVID-19 on children with CF and their lung function. The influence of potential protective mechanisms needs to be further explored to improve the quality of life in these children to enable them to lead healthy and prosperous lives during the current COVID-19 or even future respiratory pandemics.

## Ethical Approval and Consent to Participate

This study was approved by the Institutional Review Board of the Medical Research Center at Hamad Medical Corporation under protocol number (MRC-01-22-604) on 17^th^ of October 2022. All participants respected confidentiality and privacy. The study was conducted in full conformance with the principles of the “Declaration of Helsinki,” Good Clinical Practice, and within the laws and regulations of the Ministry of Public Health in Qatar. As a retrospective chart review study, the Institutional Review Board of the Medical Research Center at Hamad Medical Corporation waived the need for informed consent.

## Consent for Publication

Not applicable.

## Availability of Data and Materials

The data presented in this study are de-identified and available upon request from the corresponding author.

## Competing Interests

The authors declare no conflicts of interest.

## Funding

This study received no external funding.

## Author Contributions

Conceptualization was by KE and MA. SH wrote the original draft of the manuscript. KE and HK participated in patient management and critically revised the manuscript. All authors have read and approved the final version of the manuscript.

## Acknowledgments

Not Applicable.

## Figures and Tables

**Table 1.** Baseline Demographic Data.

	N (%)
**Gender**	
Male	4 (50%)
Female	4 (50%)
**CFTR mutation/Pancreatic status**	
I1234V mutation/Pancreatic sufficient	7 (87.5%)
3129del4 mutation/Pancreatic insufficient	1 (12.5%)
**Vaccination Status of COVID-19 vaccine before infection**	
Vaccinated	3 (37.5%)
Not vaccinated	5 (62.5%)
**Modality of COVID-19 diagnosis**	
Polymerase chain reaction (PCR)	5 (62.5%)
Rapid antigen testing (Ag)	3 (37.5%)
	**Median (Range)**
**Age at the time of infection (Years)**	11 (2-18)
**Body mass index (BMI) Z-score**	0.08 (-1.95 - 1.64)
**Baseline FEV1 (%Predicted) (N: 7)**	91 (78-107)

N (%): Sample size (%); CFTR: Cystic Fibrosis Transmembrane Conductance Regulator; COVID-19: Coronavirus disease 2019; FEV1: Forced Expiratory Volume in 1 second.

**Table 2.** Summary of patients ’demographics, presentation, and post-COVID-19 microbiological and radiological data and outcome.

Case	Age at COVID-19 infection (Years)	Gender CFTR mutation	Pancreatic Status	Baseline FEV1 (%Predicted)	Dornase alpha	COVID-19 Vaccination Before infection	Presenting Symptoms	Hospitalization (Number of days of hospitalization)	Antibiotics
1	18	Female	I1234V	Sufficient	90	No	Yes	URTI	No	No
2	18	Female	I1234V	Sufficient	78	Yes	Yes	None	No	No
3	16	Male	I1234V	Sufficient	93	No	Yes	None	No	No
4	11	Female	I1234V	Sufficient	91	Yes	No	Fever, URTI, Cough	Yes (3 days)	Yes
5	11	Male	I1234V	Sufficient	107	No	No	URTI, Cough	No	No
6	10	Male	I1234V	Sufficient	82	No	No	Fever, URTI, Cough	No	No
7	9	Male	c.2997-3000 ATTA	Insufficient	103	No	No	URTI, Cough	No	Yes
8	2	Female	I1234V	Sufficient	NA	No	No	Fever, URTI, Cough	Yes (10 days)	Yes

**Table:** 

Associated infection with COVID-19 infection	Bacterial Colonization (Pre COVID-19 infection)	Respiratory Culture Post COVID-19 infection	Chest CT findings at baseline (Before COVID-19 infection)	Chest CT findings (Post COVID-19 infection)	Outcome
None	MSSA	MRSA, PsA, NTM	Central cylindrical bronchiectasis	NA	Full recovery
None	MSSA	Not done	Central cystic bronchiectasis	NA	Full recovery
None	MSSA, NTM	MSSA	Central cystic bronchiectasis	NA	Full recovery
Group A Streptococcus	MSSA	MSSA, NTM	Central cystic bronchiectasis	Extended lobar involvement	Full recovery
None	MSSA	MSSA	None	NA	Full recovery
None	MRSA	MRSA	Peripheral cylindrical bronchiectasis	Extended lobar involvement +Varicoid bronchiectasis	Full recovery
None	MRSA	MRSA	Mild bronchial dilatation. No bronchiectasis	NA	Full recovery
Parainfluenza Chlamydia pneumoniae	PsA, MSSA	MRSA	None	NA	Full recovery

**COVID-19:** Coronavirus disease 2019; **CFTR:** Cystic Fibrosis Transmembrane Conductance Regulator; **FEV1:** Forced Expiratory Volume in 1 second; **CT:** Computed Tomography; **NA:** Not applicable; **URTI:** Upper respiratory tract infection; **MSSA:** Methicillin sensitive staphylococcus aureus; **MRSA:** Methicillin-resistant staphylococcus aureus; **PsA:** Pseudomonas aeruginosa; **NTM:** Non-tuberculosis mycobacterium.
